# Bruton’s Tyrosine Kinase Inhibitors: A New Generation of Promising Agents for Multiple Sclerosis Therapy

**DOI:** 10.3390/cells10102560

**Published:** 2021-09-27

**Authors:** Antonio García-Merino

**Affiliations:** Neuroimmunology Unit, Foundation for Biomedical Research, Puerta de Hierro University Hospital, Universidad Autónoma de Madrid, Majadahonda, 28222 Madrid, Spain; jgmerino@madrid.salud.org

**Keywords:** Bruton’s tyrosine kinase, multiple sclerosis, B cells, BTK inhibitors

## Abstract

B cells play a central role in the pathogenesis of multiple sclerosis (MS), as demonstrated through the success of various B cell-depleting monoclonal antibodies. Bruton’s tyrosine kinase (BTK) is a critical molecule in intracellular signaling from the receptor of B cells and receptors expressed in the cells of the innate immune system. BTK inhibitors may be a non-cell-depleting alternative to B cell modulation. In this review, the structure, signaling, and roles of BTK are reviewed among the different inhibitors assayed in animal models of MS and clinical trials.

## 1. Introduction

Multiple sclerosis (MS) has classically been considered an autoimmune disease of the central nervous system (CNS) with a degenerative component that becomes increasingly more evident as the disease progresses. Both the adaptive and the innate arms of the immune system are involved in the pathogenesis of MS. Even though immunoglobulin abnormalities found in the cerebrospinal fluid (CSF) are the most conspicuous immunological finding, it was still believed that anomalies of the humoral response did not have the same pathogenetic relevance as those of the cellular response. Knowledge derived from experimental autoimmune encephalomyelitis (EAE) has also contributed to the view of MS as a T-cell disease. Strong support against this view was provided by the results of a phase II clinical trial with rituximab, a monoclonal antibody depleting CD20+ B cells in patients with relapsing-remitting MS who showed a rapid and profound response in clinical and magnetic resonance imaging (MRI) parameters [1]. However, another anti B-cell trial using a fusion protein, atacicept—which binds to the cytokines BlyS and APRIL, and is involved in B-cell maturation, differentiation, and survival—had to be prematurely terminated due to disease reactivation. This failure revealed the complexity of the pathogenic role of B cells in MS [2]. Two phase III trials with ocrelizumab and ofatumumab further confirmed the beneficial effect of B cell depletion with anti-CD20+ monoclonal antibodies [3,4].

B cells play highly complex roles in physiological and pathological situations. Immature autoreactive cells are removed from the total B cell repertoire at two central and peripheral tolerance checkpoints, a process that may be relevant in autoimmune disorders such rheumatoid arthritis [5]. In MS, there is evidence of a defective peripheral, not central, B cell tolerance checkpoint [6]. In addition, as precursors of antibody-secreting cells, B cells are specialized antigen-presenting cells (APCs) for T cells [7], which produce pro- and anti-inflammatory cytokines, and have regulatory functions [8]. In MSB cells can form ectopic lymphoid follicles in the meninges [9]. Those roles have been detailed elsewhere [10].

Even if the direct effect on antibodies cannot account for the rapid effect of anti-CD20+ drugs, they have an undisputed, although ill-defined, pathogenetic role. The presence of immunoglobulin electrophoretic abnormalities in the CSF, appearing as IgG oligoclonal bands (OB) in isoelectric focusing, is an important diagnostic biomarker. From a clinical standpoint, the presence of lipid-specific IgM OB suggests a more severe disease course [11]. In vitro experiments have demonstrated demyelination and axonal damage after the addition of IgG from patients [12]. To date, no conclusive self-antigen has been recognized by the OB; however, these antigens connect with peripheral B cell responses, being part of an immune response that occurs at both sides of the blood–brain barrier (BBB) [13]. In MS, presence of immunoglobulins, anti-myelin antibodies, and complement at sites of active demyelination, characterize the immunopathological type II described by Lucchinetti et al. [14].

B cells are potent APCs that can drive T-cell mediated autoimmunity [7]. In EAE, they have a critical pathogenetic role, dependent on MHC II, and independent of their humoral response [15]. Memory B cells from MS patients elicited CD4+ T cell proliferation in response to myelin basic protein (MBP) and myelin-oligodendrocyte glycoprotein (MOG), a finding not reproduced with memory B cells from healthy donors [16]. B cells produce pro-inflammatory cytokines, such as lymphotoxin alpha, IL6, IL2, IL17, and tumor necrosis factor-alpha (TNF-α) [17]. B cells from MS patients secrete more IL6 than healthy controls [18] and abnormally secrete TNF-α and lymphotoxin when activated with interferon-γ or the pathogen-associated CPG-DNA [19]. On the other hand, B cells can act as regulators in autoimmunity by different anti-inflammatory cytokines, mostly IL10 [8]. Mice with IL10-deficient B cells develop a more severe EAE [20]. In MS patients, B cells produce a decreased average secretion of IL10 [21]. Ectopic follicle-like aggregates containing B cell and plasma cells may appear in meninges from patients with secondary progressive MS [22]; however, early MS [23] can also be associated with cortical demyelination [23,24]. In the CNS of progressive MS, antigen-experienced B cell clones obtained from meninges were related to clones that were isolated from inflammatory infiltrates, normal-appearing white matter, and CSF [25]. Importantly, the immunological abnormalities of B cells can be detected on both sides of the BBB—as demonstrated by the sequencing of IgG heavy chain variable region genes (IgG-VH) of samples of CSF and blood processed in parallel—indicating the exchange of these cells across the BBB [26].

Currently, B cells are considered fundamental players in the pathogenesis of MS [27].

Based on the fundamental role of B cells in MS pathogenesis, and considering the profound therapeutic impact of anti-CD20+ monoclonal antibodies, other anti-B cell alternatives have been explored to circumvent the problems associated with chronic B-cell depletion, such as humoral deficiency [28]. Bruton’s tyrosine kinase (BTK) has emerged in recent decades as a critical target for diseases in which B cells have a major involvement, as is the case for several hematologic malignancies. Additionally, experimental models of human autoimmune diseases have revealed that BTK is a target of the utmost importance to cancel B cell pro-inflammatory functions without the risks associated with cell depletion.

## 2. Review

### 2.1. Bruton’s Tyrosine Kinase (BTK)

In 1952, Ogden Bruton described an inherited condition in infants and children, characterized by repeated bacterial infections and the absence of serum antibodies [29]. This condition, named X-linked agammaglobulinemia (XLA), required the periodic replacement of immunoglobulins. In 1993, a gene mapping the XLA locus was identified, this gene was a member of the Src family of proto-oncogenes that encode protein-tyrosine kinases [30]. The encoded protein was later named Bruton’s tyrosine kinase (BTK). In XLA patients, the protein mutation caused a block of the B cell development at the pre-B stage in the bone marrow [31]. It was found that BTK is not only a key molecule in the signal transduction of B cells but also in other signaling pathways of B cells and myeloid cells. Clinical interest in this molecule increased exponentially after the discovery of its role in B cell malignancies and autoimmune disorders, and the availability of small molecule inhibitors for those diseases also increased.

As a tyrosine kinase, BTK belongs to a group of enzymes that catalyze the incorporation of phosphate groups from ATP into tyrosine residues. The resulting phosphorylated proteins display a wide array of cell functions, activating or inactivating other proteins in cascades. According to their cell location, tyrosine kinases are divided into two groups: the receptor group, which includes 58 types of tyrosine kinases anchored at the cell membrane; and the non-receptor tyrosine kinases (nRTKs) group, comprising 32 types, localized in the cytoplasm; most nRTKs are related to the signal transduction by lymphocyte antigen receptors [32]. BTK is a member of the TEC family of protein-tyrosine kinases, belonging to the group of nRTKs, and composed of five members. They play critical roles in the growth and differentiation of blood cells, being part of the intracellular signaling mechanisms [33].

**Figure 1:**
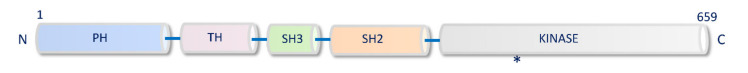
Schematic representation of the 659 amino acid BTK structure. BTK has five domains. The pleckstrin homology domain (PH) has phospholipid binding ability, which allows for the recruitment of BTK from the cytosol to the plasma membrane. The Tec homology domain (TH) plays an essential role in BTK stabilization. The Src domains (SH3 and SH2) are involved in protein–protein interactions. The kinase domain is the catalytic part of the protein. Each domain interacts with different signaling molecules. * Indicates the cysteine-481 residue position in the kinase domain, which is the site for covalent binding of the BTK irreversible inhibitors.

BTK is a 659 amino acid enzyme with the following five protein domains from the N-terminal: pleckstrin homology domain (PH), TEC homology domain (TH), Src homology domains 2 (SH2) and 3 (SH3), and the catalytic kinase C-terminal domain. Each domain interacts with different proteins that are also critical in intracellular signaling [34] (Figure 1). BTK is involved in the BCR downstream signaling cascade and in the signaling of other receptors, such as Toll-like receptors (TLR), chemokine receptors, and Fc receptors. Additionally, BTK is expressed in myeloid cells, such as macrophages [35], dendritic cells [36], microglia [37], or mast cells [38]. BTK levels were thought to be absent or undetectable in NK cells or T cells; however, this kinase is required for NK activation [39] and may be upregulated in T cells from patients with aplastic anemia [40].

### 2.2. B Cell Receptor and BTK Signaling

Studies in mouse models, known as *xid* (X-linked immunodeficiency), revealed the central role of BTK in the BCR signaling, differentiation, and survival of B cells [41]. Further experiments with mature B cells using BTK inhibitors demonstrated the involvement of BTK in B cell malignancies and models of autoimmune diseases [42].

**Figure 2:**
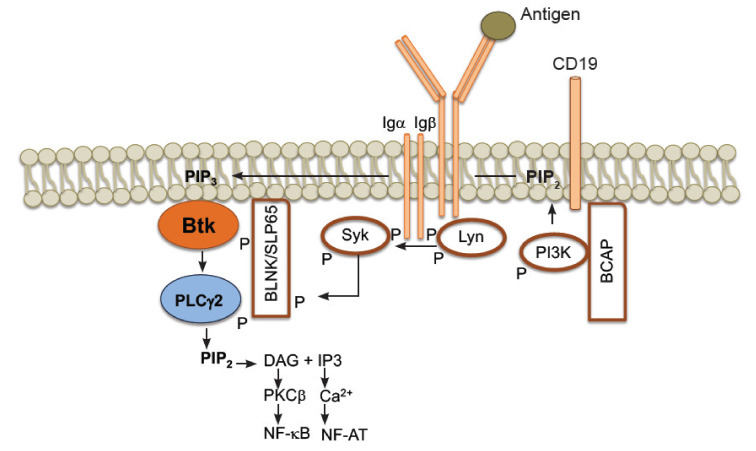
Schematic representation of role of BTK in signaling after antigen binding to the B cell receptor (BCR). Antigen binding triggers a cascade of signaling events, which leads to BTK translocation from the cytosol to the cell membrane through their union to phosphatidylinositol (3,4,5)-trisphosphate (PIP_3_). BTK phosphorylates phospholipase C gamma 2 (PLCγ2), cleaves phosphatidylinositol 4,5-bisphosphate (PIP2), and generates two second messengers: inositol 1,4,5-triphosphate (IP3) and diacylglycerol (DAG). This activates pathways, leading to the nuclear factor of activated T cells (NFAT) and nuclear factor kappa B (NFkB). (Figure taken from Román-García [43], with permission).

The B-cell receptor comprises a membrane immunoglobulin molecule of one isotype with two light chains and two heavy chains, bound by disulfide bridges and a disulfide-linked heterodimer, called Igα/β (CD79A/B), which is non-covalently associated with transmembrane tails. These tails contain immunoreceptor tyrosine-based activation motifs (ITAMs), which are required for signal transduction [43]. Upon antigen recognition by the BCR, the ITAMs of Igα/β are phosphorylated by Lyn, a member of the Src kinase family that also phosphorylates the tyrosine residue of the intracellular tail of coreceptor CD19. This phosphorylation activates and binds phosphatidylinositol 3-kinase (PI3K) to the B-cell adapter (BCAP) [44]. BTK is recruited from the cytosol to the plasma membrane by phosphatidylinositol (3,4,5)-trisphosphate (PIP_3_) through the PH domain, enabling SYK to activate BTK. The Y204 residue in the Igα recruits the adapter SLP65/BLNK through the SH2 domain [45]. Active BTK phosphorylates phospholipase C gamma2 (PLCγ2), which generates two second messengers: inositol 1,4,5-triphosphate (IP3) and diacylglycerol (DAG) [46]. IP3 activates the calcium channels, allowing for the transport of the nuclear factor of activated T cells (NFAT) into the nucleus. On the other hand, DAG activates protein kinase C β (PKCβ), the pathways of mitogen-associated protein kinase (MAPK) and nuclear factor kappa B (NFκB). (Figure 2). NFAT and NFκB regulate the expression of several genes that are crucial for B cell survival and proliferation, and chemokine and cytokine expression [47] (Figure 2).

### 2.3. Functions of BTK Unrelated to the BCR

Chemokine gradients are fundamental regulators of lymphocyte circulatory patterns. Chemokine receptors are G-protein coupled with seven transmembrane domains and an intracellular domain with three protein subunits. They are present in all leukocyte types. BTK is a fundamental player in the signaling cascade, induced by the activation of these receptors; the protein subunits may modulate BTK through the activation of PI3K signaling and binding to the PH and TEC domains [48]. The chemokine receptors, CXCR4 and CXCR5, are expressed in B cells and are related to trafficking and homing. BTK and PLCg2 are responsible for the chemokine-induced migration mediated by integrins [49]. The inactivation of BTK may alter the expression of the chemokine receptor, resulting in impaired migration and the homing of B cells, as seen in patients with chronic lymphatic leukemia (CLL) that were treated with ibrutinib [50]. The impairment of B cell traffic may have therapeutic relevance for MS.

Toll-like receptors (TLRs) play a critical role in innate immune responses. They are expressed in B cells and myeloid cells and belong to a family of transmembrane proteins that recognize conserved molecules from microorganisms. BTK is involved in the interactions with proteins downstream of TLR signaling. These interactions may modify activation, proliferation, antibody secretion, class switch, and pro-inflammatory cytokine secretion, leading to the induction of NFkB, activator protein 1 (AP-1), and interferon regulatory factor 3 [48].

Granulocyte-macrophage stimulating factor (GM-CSF) is a family of glycoproteins with many functions on hematopoietic cells and important roles in innate and adaptive immunity, with an increasingly more evident role in autoimmune disease, including in MS [51]. BTK inhibition may skew the phenotype of activated macrophages from the pro-inflammatory M1 phenotype to the M2 anti-inflammatory phenotype [52].

Fc receptors are crucial for innate cell functions. BTK is involved in the signaling of IgG-specific Fc receptors (FcgR), with either activating or inactivating effects. In addition, it also participates in the signaling of various interleukin receptors and CD40 [41].

BTK is also implicated in integrin activation after BCR ligation, controlling adhesion, mediated by VLA-4 to VCAM-1 [53]. In addition, BTK regulates the interaction between B cells and APCs, which is termed the immune synapse [54].

Inflammasomes are multiprotein complexes that regulate the maturation of pro-inflammatory cytokines such as IL1b or IL18 with significant pathophysiological roles in several conditions, including MS [55]. As BTK is a regulator of NLRP3 [56], the pharmacological inhibition of this kinase using small molecules may be an alternative to target the NLPR3 inflammasome [57].

### 2.4. Role of BTK in Myeloid Cells

In addition to its capital role in B cells, BTK is also involved in the functions of other cell lineages that are pathogenetically relevant for MS, such as monocytes and macrophages, dendritic cells, and microglia. Macrophages are derived from circulating monocytes that differentiate their inflammatory surroundings. They present antigen to T cells and have critical phagocytic capacity and cytokine secretion. The phagocytic ability of the macrophages is mediated by FcγR and the cytokine secretion of TNFα and IL1b by TLR4. Signaling after the stimulation of those receptors involves BTK [41]. BTK inhibition by ibrutinib suppressed the FcγR-mediated secretion of TNFα but did not interfere with phagocytosis [53]. Bacterial lipopolysaccharide (LPS) is a potent stimulator of TLR, generating a downstream signal that is mediated by BTK. After LPS stimulation, experiments with BTK-/- mice demonstrated the role of BTK in the polarization of M1/M2 macrophages [58]. In patients with CLL, ibrutinib modified the function of the monocyte and macrophages, increasing the expression of M2 markers [59]. In monocytes from healthy volunteers, the irreversible inhibitor, evobrutinib (see below), skewed macrophage polarization towards the M2 anti-inflammatory phenotype [52].

Dendritic cells are APCs to T lymphocytes, along with B cells and macrophages. They represent a bridge between innate and adaptive immunity, have a pathogenetic role in immune diseases, such as MS, and are being used for tolerizing approaches in MS [60]. BTK is involved in the activation and differentiation of dendritic cells; however, compared to B cells, BTK functions are not as well-defined [41].

Microglia are phagocytic cells which reside in the CNS, derived from progenitor cells in the embryonic yolk sac that migrate into the CNS. They constitute a fundamental part of the innate immunity in the CNS and express BTK [61]. BTK modulates microglial phagocytosis in murine models of Alzheimer’s disease [62]. Targeting microglia with small-molecule BTK inhibitors (BTKi) could develop into a therapeutic tool of great interest for MS and neurological disorders in which microglia have a pathogenic implication.

Mast cells are also components of the innate arm of the immune system. They are residents in the meninges and their role in MS pathogenesis has recently been recognized; they have disease-promoting or protecting effects on EAE [63]. BTK is greatly expressed in mast cells and is involved in the signaling of the high-affinity IgE receptor. The activation of mast cells may be blocked by BTKi [64].

### 2.5. BTK Inhibitors

Soon after the discovery of BTK, the search for anti-leukemic agents led to the investigation of BTKi. LFM-A13 was the first BTKi to be synthesized [65], as well as the first anti-leukemic agent targeting BTK. During the following years, more selective inhibitors were designed. Ibrutinib, an irreversible BTKi, was synthesized in 2007 [66]. This small molecule represents a major advancement in the therapy of B cell malignancies. In 2013, the FDA approved ibrutinib for mantle cell lymphoma and CLL, and years later, Waldenström’s macroglobulinemia. The success with ibrutinib and the ample therapeutic possibilities for diseases with a pathogenetic role of B cells fostered the development of new, more selective, and better-tolerated inhibitors.

BTKi are small-molecule agents. Small molecules have several advantages for biological modulation, compared to large molecules, such as oral dosage, intracellular targeting, and lower manufacturing costs [67]. For MS, the size of these molecules may be another advantage in terms of their ability to cross the BBB, as reported with some inhibitors.

Depending on the union with BTK, inhibitors are classified as irreversible or reversible. Irreversible inhibitors usually bind to cysteine residue 481 of the kinase domain through covalent bonds. As cysteine-481 is critical for ATP binding, which is required for catalytic activity, the union with inhibitors determines the suppression of signaling downstream of BTK [68]. Ibrutinib is a potent, first-in-class, small-molecule inhibitor. Significant adverse events, such as cardiac arrhythmias, diarrhea, bleeding, infection, arthralgias, and hypertension [69], preclude ibrutinib from being used for autoimmune disorders. These unwanted complications are due to off-target effects—non-specific interactions with other kinases, such as EGFR, ITK, JAK3, HER2, and TEC. New generations of irreversible inhibitors, such as acalabrutinib, have increased selectivity and reduce the level of activity on other kinases [68]. Ten kinases in humans have an equivalent cysteine residue in their active site: this circumstance may be associated with unwanted off-target inhibition [70].

Reversible inhibitors do not bind to cysteine-481 and are good alternatives for persons with B-cell malignancies who do not respond to irreversible inhibitors due to BTK cysteine-481 mutations [71]. Reversible inhibitors non-covalently bind to different BTK-specific pockets with hydrogen bonds, ionic bonds, or hydrophobic forces [68]. Weak binding decreases potency, but also toxicity and risks associated with chronic use. These factors make reversible inhibitors good candidates for autoimmune diseases. However, progress in the development of BTKi has been more rapid with irreversible inhibitors.

It is important to stress that, contrary to X-linked agammaglobulinemia, the pharmacological inhibition of BTK does not cause significant decreases in immunoglobulin levels. This is, in part, due to the fact that these inhibitors act on the kinase domain of the BTK molecule, leaving other domains untouched [72]. The treatment of B cell malignancies with ibrutinib did not induce a reduction in total immunoglobulin levels [73]. In a study in patients participating in a phase II clinical trial with evobrutinib for MS, B cell depletion, or a significant decrease in the immunoglobulin levels in blood, was not detected after 48 weeks [74]. However, in a clinical trial with ibrutinib for systemic lupus erythematosus, ibrutinib caused a reduction in the levels of some autoantibodies [75].

### 2.6. BTKi in EAE

The efficacy of tyrosine kinase inhibitors in animal models of MS was demonstrated by Crespo et al. [76] In the actively induced EAE of C57BL6 mice inoculated with the peptide 35-55 of MOG (MOG_35–55_), three different tyrosine kinase inhibitors (sorafenib, imatinib, and GW2580) ameliorated the disease course, either before or after the appearance of the clinical signs. In parallel, the CNS of treated animals exhibited significantly lower levels of inflammation. Sorafenib and GW2580 suppressed TNF production by macrophages, and imatinib reduced astrocyte proliferation through PDGF induction. None of these inhibitors had selectivity on BTK.

Tyrphostin AG126, a member of a family of protein tyrosine kinase inhibitors, is a BTKi with other complex effects. Treatment with tyrphostin AG126 before or after disease onset improved clinical scores in the EAE of C57BL6 mice inoculated with MOG_35–55_, reduced inflammatory infiltration in the spinal cord and decreased myelin damage, Th17 cell differentiation, and microglial activation. Among the observed immunological effects induced by this compound, B and T cell changes, and the modulation of microglia, were attributed to BTKi. However, BTK inhibition was not sufficient to explain the full spectrum of results, including interference with signaling from TLR, independent of BTK [37].

A recent report showed the immunological effects of BTK selective inhibition in EAE [77]. Evobrutinib was used to treat C57BL6 mice with MOG_1–117_-induced EAE; MOG_35–55_ TCR transgenic mice were also used. Evobrutinib ameliorated clinical and histological EAE, administered prior to disease onset; the number of B-cell infiltrates in the CNS was markedly reduced with a relative decrease in T cells. Of note, the drug inhibited the expression of molecules involved in B cell antigen presentation, impairing their ability to generate encephalitogenic T cells. On B cells, evobrutinib greatly inhibited the activation and differentiation mediated by TCR. In separate experiments with human B cells from healthy volunteers and MS patients, there were no differences in BTK expression in the B cell subsets studied, in contrast with what was found in rheumatoid arthritis and in a fraction of patients with Sjögren syndrome, where BTK expression was increased in B cells [78].

Martin et al. analyzed the possible effect of BTK inhibition in experimental models of demyelination/remyelination, using MBP-GFP-NTR *Xenopus* transgenic tadpoles and mouse cerebellar organotypic slice cultures [61]. Transgenic tadpoles exposed to metronidazole suffered oligodendrocyte damage and demyelination, followed by spontaneous remyelination. They induced demyelination in cerebellar slice cultures with lysophosphatidylcholine. Immunohistochemistry revealed that most cells expressing BTK were microglia, and BTK expression increased sharply in demyelinated slices. BTK inhibition with evobrutinib increased the rate of remyelination in demyelinated slice cultures and transgenic tadpoles [61] (Table 1).

### 2.7. BTKi in MS

Tolebrutinib is an irreversible inhibitor, also known as SAR442168, PRN2246, or BTK’168, which covalently binds to cysteine-481 in the kinase domain. In healthy volunteers, 2 h after oral administration, a lumbar puncture revealed significant concentrations of this molecule in the cerebrospinal fluid, exceeding cell-based IC_90_ [79]. In a phase IIb dose-finding study in relapsing MS (RMS) with particularly active disease, the primary objective of which was the reduction in new active brain lesions (NCT03889639), tolebrutinib 60 mg achieved favorable results after 12 weeks of treatment [80]. Tolebrutinib has four ongoing phase III trials targeting the three main clinical forms of MS. RMS is assayed in two identical trials of tolebrutinib versus teriflunomide, GEMINI 1 and 2, with 900 participants each. Non-relapsing secondary progressive MS (SPMS) is the object of a trial versus placebo (HERCULES) with 1290 participants. Primary progressive MS (PPMS) is currently being studied in another trial of 990 patients with tolebrutinib versus placebo (PERSEUS).

Fenebrutinib (GDC-0853) is an entirely different inhibitor that does not bind to cysteine-481. Instead, it forms hydrogen bonds with lysine-430, methionine-477, and aspartate-539 residues of the kinase domain [81]. This reversible inhibitor does not inhibit EGFR or ITK, another member of the TEK kinase family that drives T-cell receptor signaling [82]. Fenebrutinib induces a conformational change that inactivates BTK, preventing the activation of downstream targets. In phase II clinical trials, fenebrutinib was tested for systemic lupus erythematosus, rheumatoid arthritis, and chronic urticaria [81]. Concerning MS, three phase III clinical trials are ongoing: one with PPMS, including 946 patients, which will analyze the drug (or placebo) versus ocrelizumab (or placebo) (FENtrepid). RMS will be the object of two identical trials, including 734 patients, each with fenebrutinib versus teriflunomide (FENhance).

Evobrutinib is an irreversible inhibitor, binding covalently to cysteine-481 through a warhead that enhances selectivity over other kinases. Interaction with the gatekeeper threonione-474 increases selectivity, as only 10% of human kinases have threonine as a gatekeeper residue [83]. Compared to ibrutinib, these characteristics suggest that evobrutinib is less potent but more selective, with a potential profile of fewer off-target associated events. In murine models of systemic lupus erythematosus and rheumatoid arthritis, evobrutinib exhibited robust clinical and histological efficacy by acting on B cells and innate immunity cells [84].

To date, evobrutinib is the BTKi for MS with more detailed results elucidated through a phase II, placebo-controlled clinical trial that was recently published [85]. A total of 267 patients with RMS were randomly assigned to five groups: placebo, evobrutinib at three doses (25 mg once daily, 75 mg once daily, and 75 mg twice daily), and an open group that was treated with dimethyl fumarate as a reference. After 24 weeks, patients on placebo were switched to 25 mg of evobrutinib daily, and the rest of the groups continued with the same medication for an additional 24 weeks. The primary endpoint was the cumulative number of gadolinium-enhanced lesions in T1-weighted MRI at weeks 12, 16, 20, and 24. Secondary endpoints were marked by the change in the annualized relapse rate and in the expanded disability status scale (EDSS) score from baseline and safety. Only evobrutinib at 75 mg daily significantly reduced MRI lesions from weeks 12 through to 24, compared to the placebo. Of note, 75 mg evobrutinib twice daily failed to demonstrate efficacy. Evobrutinib did not have any significant effect on relapse rate or disability progression at any dose. Regarding adverse events, the higher doses of the drug (75 mg once and 75 mg twice daily) were associated with a higher frequency of aspartate aminotransferase, alanine aminotransferase, and lipase elevation, leading to trial discontinuation in some patients. Cardiac arrhythmias, bleeding, or hypertension, frequently seen with the irreversible BTKi, ibrutinib, were not reported. Currently, evobrutinib is being assayed in two identical phase III trials in RMS, Evolution 1 and 2, with 930 patients each.

Orelabrutinib is a potent, second-generation, irreversible BTKi, developed for B cell malignancies and autoimmune diseases, including MS [86]. A phase II randomized, double-blind clinical trial for 160 relapsing–remitting MS patients is ongoing.

Table 2 shows all clinical trials with BTKi for MS currently running.

## 3. Discussion

For B cell malignancies, BTK inhibition by small molecules represents a major therapeutic advance. The enthusiasm for these types of drugs has expanded toward the field of autoimmune diseases with B cell-dependent pathogenesis. Experience with BTKi has developed with experimental models of several human autoimmune diseases, but clinical advances have been much more limited. For the time being, clinical trials with different BTKis are in various phases of development, but none of them has gained the approval of regulatory agencies.

Trying to decipher precise mechanisms of BTKi in MS may be a challenging task, not only because of the complexity of the enzyme involved in a constellation of intracellular functions, but also because it is premature due to the lack of clinical experience. Therefore, most of the currently available knowledge is derived from experimental models.

As shown with in vitro and in vivo experiments, the inhibition of BTK affects nuclear factors that are essential for maturation, proliferation, and cytokine production by B cells. In EAE, B cell–T cell interactions are affected, leading to a strong reduction in the ability to activate naïve T cells that promote encephalitogenic T cells, and a marked decrease in pro-inflammatory cytokine secretion, as clearly demonstrated with evobrutinib [77]. Interference with B cell maturation may determine a diminished generation of new pathogenic B cells. In MS, it remains to be seen if BTKis serve to eliminate autoreactive B cells, more dependent on BTK for survival than normal B cells [72], and the extent to which they can modify the different B cell types, particularly memory B cells, which are considered the major targets for immunotherapy [87].

In murine models of lupus, BTK expression is increased and facilitates the generation of autoantibodies by promoting plasma cell differentiation from pathogenic B cells; BTKi may block the formation of these antibodies [88]. Clinical trials with ibrutinib in lupus patients reported a reduction in some autoantibodies [75]. BTK overexpression is not detected in MS [78]; therefore, the mechanism that associates increased BTK expression with autoantibodies seems unlikely. In fact, evobrutinib did not cause a significant decrease in total immunoglobulin levels [74]. Nonetheless, one cannot rule out the possible impact of chronic BTKi therapy on IgG deposition in the CNS.

Alteration in the downstream signals generated from the BCR appears to be the most important mechanism of action for the BTKi in B cells. In addition to the BCR, interference with the signaling of other receptors present in B cells and cells of the innate immune system may significantly contribute to the therapeutic effect. Chemokine receptors CXCR4 and CXCR5, related to migration and homing, may be a contributing factor. The inhibition of signaling from TLR, FcgR, GM-CSFR, the control of adhesion, or the effect on the NLRP3 inflammasome are also factors to be explored in depth. In MS and other autoimmune diseases, the impact of BTKI on those receptors poses many questions on the pathogenic role of the innate immune system [57]. The expression of BTK in microglial cells, experimentally demonstrated [61,62], and the permeability of the BBB to small molecule inhibitors [79] raises the possibility of effectively modulating the activated microglia in MS patients.

Since the introduction of ibrutinib, safety has been an essential concern for developing new BTKis. A number of molecules have been synthesized in the search for BTKis with higher selectivity and fewer off-target effects that are adequate for safer long-term use. In theory, non-covalent reversible inhibitors would be more appropriate for autoimmune disorders, at the cost of lower efficacy. Published experience in a phase II trial with the irreversible inhibitor, evobrutinib, suggested that it has an acceptable safety profile. Only the long-term exposure of MS patients to both types of BTKis in phase III clinical trials will provide a larger perspective concerning safety and tolerance.

In animal models of autoimmune diseases, including MS, results with different BTKis have raised expectations, and time will show whether the efficacy of those models translates to human diseases [89]. In EAE, experience with evobrutinib revealed the drug’s central mechanism of action, inhibiting the activation of pathogenic T cells, a mechanism shared by anti CD20+ monoclonal antibodies [77]. It is inevitable to comment on the differential effects of those antibodies versus BTKi in MS. A phase II trial with rituximab showed an almost immediate and robust cancellation of CNS inflammation, detected as soon as four weeks after the first dose, with a marked effect on relapses [1]; in contrast, only one dose of evobrutinib achieved a significant reduction on MRI lesions from weeks 12 to 24, with no effect on secondary outcomes [85]. Compared to evobrutinib, and even though direct comparisons are not feasible, evidence indicates that the anti-CD20+ monoclonal antibodies exert a level of disease control that is not reached by evobrutinib. However, regarding efficacy against MS, the available information is preliminary, making it difficult to draw conclusions on BTKi. We will only know the real impact of those compounds on MS after the completion of the currently ongoing phase III clinical trial. BTKi offers a series of good points for long-term therapy: ease of administration and access to the CNS for the effective control of inflammation and the possibility of utilization in conjunction with anti-CD20+ antibodies, not as combined therapy, as is used in B cell malignancies—in which different clinical protocols associate BTKi with rituximab (or other anti-CD20+ antibodies) [90]—but in sequence, as suggested by Torke et al. [77] Sequential use after B cell depletion with these antibodies would have the advantage of rapid inflammatory control, followed by drugs that inactivate B cells without significant effects on immunoglobulin levels.

## 4. Conclusions

Over the last three decades, immune therapy for MS has passed important milestones, effectively controlling clinical and MRI activity, even in patients with significantly active disease, slowing the accumulation of disability and delaying entry into the secondary progressive phase [91,92]. Despite modest advances with SPMS or PPMS, progression has been a stumbling block for therapy. Progression in MS is complex and is thought to depend on different factors, some of which are not well understood. Persistent inflammation within the CNS stands out as one of the candidate factors. Inflammation accompanies myelin damage alongside the evolution of the disease; the composition of the inflammatory infiltrate includes CD8+ T lymphocytes, B cells, activated microglia, and macrophages [93]. In chronic stages, BBB permeability is restored, preventing the entry of therapeutic agents. Compartmentalized or “trapped” inflammation [94] in the CNS has become an important challenge for therapy, and it may be a major contributor to myelin and axonal damage. Additionally, the presence of ectopic B cell follicle-like structures in the meninges are related not only to cortical damage [23,24] but also to spinal cord pathology [95].

For MS, the availability of a family of new drugs that can reach therapeutic concentrations inside the CNS, counteract the inflammation driven by B cells, and modulate the critical players in innate immunity, such as macrophages and microglia, is a therapeutic promise, due to the fact that other disease-modifying drugs do not fulfill those aims. If these inhibitors effectively control persistent CNS inflammation, this could represent a great step toward the prevention of MS progression. We are currently in the preliminary—albeit exciting—stage of development, with many ongoing clinical trials hoping that the promise of BTKi will turn into reality. Intense clinical research will follow in the coming years.

## Figures and Tables

**Table 1:** Tyrosine kinase inhibition in experimental models of multiple sclerosis.

Inhibitors	BTK Selectivity	Model	Effects	Ref.
Sorafenib, Imatinib, GW2580	No	EAE C57BL6 mice	Improved disease course. Decreased CNS inflammation. Reduced TNF production and astrocyte proliferation.	[76]
Tyrphostin AG126	Yes Other effects	EAE C57BL6 mice	Improved evolution. CNS inflammation, myelin damage, Th17 differentiation, and microglial activation decreased.	[37]
Evobrutinib	Yes	EAE C57BL6 mice; transgenic mice	Clinical improvement. Inhibition of B cell activation and maturation. Decreased pro-inflammatory cytokine secretion. Marked decrease in the number of B cell infiltrates; reduction in the number of T-cell infiltrates. Impaired ability to generate encephalitogenic T cells.	[77]
Evobrutinib	Yes	*Xenopus* transgenic tadpoles; mouse cerebellar organotypic cultures	Increased remyelination in demyelinated slice cultures and transgenic tadpoles.	[61]

**Table 2:** Bruton’s tyrosine kinase inhibitors currently assayed in clinical trials for multiple sclerosis.

Product	Type of BTKi	Sponsor	ClinicalTrials Gov Identifier	Phase	Type of Trial	Patients	Start Date	Estimated Completion Date
**Fenebrutinib**	Non-covalent, reversible	Hoffmann-La Roche	NCT04544449	III RDB	Fenebrutinib (or placebo) vs. ocrelizumab (or placebo) 1:1 (FENtrepid)	946 PPMS	2020	2028
**Fenebrutinib**	Non-covalent, reversible	Hoffmann-La Roche	NCT04586023	III RDB	Fenebrutinib vs. teriflunomide 1;1 (FENhance)	734 RMS	2021	2024
**Fenebrutinib**	Non-covalent, reversible	Hoffmann-La Roche	NCT04586010	III RDB	Fenebrutinib vs. teriflunomide 1;1 (FENhance)	734 RMS	2021	2024
**Tolebrutinib**	Covalent, Irreversible	Sanofi/Principia	NCT04458051	III RDB	SAR442168 (tolebrutinib) vs. placebo (PERSEUS)	990 PPMS	2020	2024
**Tolebrutinib**	Covalent, Irreversible	Sanofi/Principia	NCT04410978	III RDB	SAR442168 (tolebrutinib) vs. teriflunomide GEMINI1	900 RMS	2020	2023
**Tolebrutinib**	Covalent, irreversible	Sanofi/Principia	NCT04410991	III RDB	SAR442168 (tolebrutinib) vs. teriflunomide GEMINI2	900 RMS	2020	2023
**Tolebrutinib**	Covalent, irreversible	Sanofi/Principia	NCT04411641	III RDB	SAR442168 (tolebrutinib) vs. placebo (HERCULES)	1290 SPMS	2020	2024
**Evobrutinib**	Covalent, irreversible	Merck KGaA	NCT04338022	III RDB	Evobrutinib vs. teriflunomide (EvolutionRMS 1)	930 RMS	2020	2026
**Evobrutinib**	Covalent, irreversible	Merck KGaA	NCT04338022	III RDB	Evobrutinib vs. teriflunomide (EvolutionRMS 2)	930 RMS	2020	2026
**Orelabrutinib**	Covalent, irreversible	Beijing InnoCare Pharma Tech Co., Ltd.	NCT04711148	II RDB	Orelabrutinib, three doses vs. placebo at 1:1:1:1 ratio	160 RRMS	2021	2024

Legend to table. Clinical trials with BTK inhibitors for multiple sclerosis in 2021. RDB: randomized double-blind. PPMS: primary progressive multiple sclerosis. SPMS: secondary progressive multiple sclerosis. RMS: relapsing multiple sclerosis. RRMS: relapsing–remitting multiple sclerosis.

## Data Availability

Not applicable.
